# Better with GPs as managers? – Variation in perceptions of feedback messages, goal-clarity and performance across manager´s in Swedish primary care

**DOI:** 10.1186/s12913-023-09586-2

**Published:** 2023-06-14

**Authors:** Anders Anell, Anna Glenngård

**Affiliations:** Department of Business Administration, Lund University School of Economics and Management, Lund, Sweden

**Keywords:** Performance management, Teamwork, Goal clarity, Audit and feedback, Primary care managers, Patient-reported performance

## Abstract

**Background:**

Primary care in several countries is developing towards team-based and multi-professional care, requiring leadership and management capabilities at the primary care practice level. This article reports findings from a study of primary care managers in Sweden, focusing variation in performance and perceptions of feedback messages and goal-clarity, depending on managers’ professional background.

**Methods:**

The study was designed as a cross-sectional analysis of primary care practice managers’ perceptions combined with registered data on patient-reported performance. Managers perceptions was collected through a survey to all 1 327 primary care practice managers in Sweden. Data about patient-reported performance was collected from the 2021 National Patient Survey in primary care. We used bivariate (Pearson correlation) and multivariate (ordinary least square regression analysis) statistical methods to describe and analyse the possible association between managers’ background, responses to survey statements and patient-reported performance.

**Results:**

Both GP and non-GP managers had positive perceptions of the quality and support of feedback messages from professional committees focusing medical quality indicators, although managers perceived that the feedback facilitated improvement work to a lower degree. Feedback from the regions as payers scored consistently lower in all dimensions, especially among GP-managers. Results from regression analysis indicate that GP-managers correlate with better patient-reported performance when controlling for selected primary care practice and managerial characteristics. A significant positive relationship with patient-reported performance was also found for female managers, a smaller size of the primary care practice and a good staffing situation of GPs.

**Conclusions:**

Both GP and non-GP managers rated the quality and support of feedback messages from professional committees higher than feedback from regions as payers. Differences in perceptions were especially striking among GP-managers. Patient-reported performance was significantly better in primary care practices managed by GPs and female managers. Variables reflecting structural and organizational, rather than managerial, characteristics contributed with additional explanations behind the variation in patient-reported performance across primary care practices. As we cannot exclude reversed causality, the findings may reflect that GPs are more likely to accept being a manager of a primary care practice with favourable characteristics.

**Supplementary Information:**

The online version contains supplementary material available at 10.1186/s12913-023-09586-2.

## Introduction

Primary care in several countries develops towards larger, team-based practices, employing a mix of professionals [1–3]. For a number of reasons, the traditional organization of stand-alone GPs, or a few collaborating GPs working in the same facility and sharing secretary and nurse resources, is no longer the norm. In several countries, policy documents favor a development towards team-oriented care, not least due to possibilities of task shifting from doctors to nurses and to provide a better work environment with improved conditions for learning [1, 4–6]. This development is not without problems. GPs and other professionals working in the primary care setting needs competence and skills adapted to teamwork. New professional roles need to be developed. These processes also need strong collective and transformational leadership at the primary care practice (PCP) level [6]. In most countries, primary care is expected to be in the driver´s seat of preventive measures, first-line contacts and when treating major chronic diseases and frail elderly with comorbidities [1, 3]. Still, limited attention has been devoted to research on leadership and management in general practice compared to management in health care in general [2], and reviews of available studies report that good quality research is scarce [7]. Doctors themselves may feel prepared for managerial roles, but they also report that combining clinical and managerial roles and skills may be difficult and/or uninteresting [2, 8].

In contrasts to traditions in most European countries, team-based primary care has been practiced for decades in Sweden. PCPs in Sweden typically consist of 40–50 staff with a mix of professionals including general practitioners (GP), registered nurses (RN) with different specialisations (e.g. diabetes, heart disease) and physiotherapists [3, 9]. The tradition of team-based primary care has facilitated a flexible use of resources, including task shifting from GPs to RNs. In fact, RNs together with other non-physician professionals at the PCPs are responsible for the majority of all primary care visits by patients.

Since 1997 it has been formally possible for non-physicians to act as managers for PCPs [10]. In case the manager has a non-physician background, there must also be an appointed clinical director (a physician) with the overall medical responsibility. The share of RNs in managerial positions has increased substantially since the late 1990s, especially among public PCPs. Already in 2008, 50% of managers in Swedish public primary care were reported to be RNs whereas 33% were GPs [11]. These proportions were confirmed in a survey in 2010 [10] and more recent studies indicate that the proportion of non-GP managers has since then increased further [12]. This development has also contributed to a changed gender mix of PCP managers, to the favour of female managers.

Arguments, not least from physician union organizations, have been that GP-managers can be associated with better PCP performance [13]. It is more common that GP-managers perform clinical work besides their management responsibilities, and this professional link and the associated legitimacy as seen from PCP staff is referred to as a possible explanation. Indeed, a recent study based on interviews noted a difference in leadership style between GP and non-GP managers in Swedish primary care [9]. Previous studies indicate that nurse PCP managers are more committed to budgetary control and objectives assigned by regions in comparison with physician managers [10, 14]. However, to our knowledge, there is no empirical research investigating the possible association between managers’ professional background and PCP performance.

This article reports findings from a survey across managers of PCPs in Sweden. Our purpose was to investigate variation in performance of PCPs with regard to manager´s professional background. We also studied variation across managers in perceptions of feedback messages and goal-clarity. Theory as well as empirical studies suggests that both goal-clarity and feedback messages have a positive relationship with performance [15–17]. In Swedish primary care, two main and parallel forms of feedback interventions can be identified [9]: one from payers/purchasers related to assigned goals and the overall quality of PCPs, and one from professional committees focusing an appropriate use of antibiotics and other pharmaceuticals. We had a particular interest in if PCP managers with different professional background perceive the quality and support of these feedback messages differently, and the potential association with PCP performance.

## The institutional setting

The Swedish decentralized healthcare system is financed and organised by 21 geographical regions [18]. Regions individually regulate the local requirements (financial, organisational and quality requirements) that PCPs have to comply with in order to receive public funding. Public and private providers operate under similar conditions. By national law, the same requirements apply to both public and private PCPs within each region, and private providers are free to set up their PCPs without geographical restrictions [19]. As individuals are entitled to a free choice of provider, PCPs need to balance objectives of responsiveness towards patients’ preferences and adherence to requirements as stipulated in contracts with the regions. Payment to PCPs mainly consists of risk-adjusted capitation and PCPs have a comprehensive financial responsibility for providing primary care to its registered patients, including use of prescription medicines [20]. GPs and other staff categories are salaried employees in both public and private PCPs and the same restrictions regarding e.g., working hours apply regardless of ownership.

*Feedback from the payer* (regions) is targeted at compliance to contractual obligations. These contracts represent minimum requirements regarding resources, facilities and quality of services. Feedback is presented to each PCP in the form of a written report and include comparison against targets and the performance of other PCPs in the region. Written feedback is provided once a year, often combined with a meeting with the PCP manager and other relevant staff. Frequently, payers use a traffic light system (green, yellow, red) to communicate deviations. Negative deviations result in warnings and requirement of actions (to be audited). In case of serious deviation, the PCP may face financial penalties or even lose their contract.

*Feedback from STRAMA* (professional committees) is targeted at clinical data and medical evidence, in particular the use of antibiotics. Feedback is presented at both the aggregate PCP level and at the individual prescriber level. Feedback is usually provided each quarter and often in group meetings facilitated by senior professionals with a clinical background. Adherence to pharmaceutical guidelines and a restrictive use of antibiotics is generally associated with benefits for both individual patients and society (avoiding antibiotic resistance). Although negative feedback from STRAMA is usually not associated with financial penalties or sanctions, variations across PCPs are transparent and GPs may feel obliged to comply in order to preserve their professional reputation.

## Method

The study was designed as a cross-sectional analysis of PCPs. We used two sources of data; a survey to all PCP managers in Sweden and results from a national patient survey (NPS) in primary care.

The survey to all 1 327 PCP managers in Sweden was used to collect data about managers’ perceptions as well as background characteristics of themselves and the PCP. The survey was distributed as a web-link via e-mail in February 2022. Two reminders in the form of a postal letter with the questionnaire attached were sent out after two and four weeks. We use responses to 21 Likert-scale statements about feedback messages, goal clarity and self-rated performance together with categorical responses to questions about the background of managers and PCPs characteristics (see Appendix 1). Statements about self-rated PCP performance were constructed to capture the task of balancing requirements from both patients and the region. Statements about performance therefore covered three dimensions: responsiveness towards patients’ preferences (one statement) and medical needs (one statement) and adherence to requirements stipulated in agreements with the region (one statement). Statements about goal clarity were constructed to consider both assigned goals from the region (one statement) and self-determined goals at the PCP level (one statement). Construction of statements reflecting dimensions of feedback messages were based on previous studies of the design of feedback messages in health-care settings [21, 22] as well as two previous qualitative studies of feedback messages in the context of Swedish primary care [3, 9]. Perceptions of feedback messages (from the region as payer and STRAMA) covered four dimensions: information about deviations (three statements), facilitation of improvement work (two statements), quality of data in terms of verifiability/timeliness and sensitivity (two statements) and whether feedback messages stimulates social interaction (one statement).

Data about patient-rated PCP performance was collected from the 2021 NPS in primary care. The 2021 survey was answered by 89 944 individuals having visited a GP in September, a response rate of 39%. Results from the NPS were released about a month prior to the distribution of our own survey. The NPS contains background questions about the respondent and 32 Likert scale questions about their experience with the care provided. The questions are sorted into different dimensions of patients’ perceptions about each PCP. Each dimension is assigned a score between 0 and 100 based on the weighted proportion of positive answers (3–5 on the 1–5 Likert scale) to the questions sorted under each dimension, where higher values indicate better performance of the PCP. The calculation of scores and dissemination of results from the NPS is administered by the Swedish Association for Local Authorities and Regions (see [23] for details on questions and composition of scores). In this study we used the published scores for the dimension “overall impression” to measure patient-rated performance. This dimension includes answers from three questions (see appendix 1). The rationale for using this dimension is that we strived to include an existing measure of patients’ overall perceptions about PCPs, rather than their views about selected quality dimensions.

To investigate differences across managers we employ two statistical methods. We use bivariate (Pearson correlation) statistical analysis to describe correlations between managers’ background and perceptions of feedback messages, goal clarity and performance. We use multivariate (OLS regression) statistical analysis to analyse the possible association between managers’ background and patient-reported performance. The score “overall impression” from the NPS was used as the dependent variable. Independent variables were composed of survey data on managers’ background and managers’ responses to statements about feedback messages, goal-clarity and self-rated performance. We included a number of control variables representing characteristics of PCPs (size, staffing situation, ownership). Finally, we included dummy variables for each of the 21 regions to strengthen the analysis and to control for socio-demographic factors. The final choice of regression models considered multicollinearity between independent variables to make statistical inferences reliable. The responses to different statements of feedback messages were highly correlated. Therefore, factor analysis (Extraction method: Maximum-Likelihood. Rotation method: Direct Oblimin with Kaiser normalization) was used to construct two new variables representing feedback from the region and STRAMA to include in the regression analysis [24]. All statistical analysis was carried out in SPSS version 22.

Our cross-sectional survey research design comes with limitations. Most importantly, results should be interpreted as correlations since this design cannot help determine causal relations. Secondly, results represent a specific point in time and cannot be used to analyse patterns over a period in time. Thirdly, a low response rate implies that it is important to be careful in generalizing the results. Finally, the number and quality of the control variables used for measuring PCP characteristics are limited. Variables representing the staffing situation of PCPs was based on managers’ perceptions, as no other comparative data was available.

## Results

### Data

Of the target population, managers of all 1 327 PCPs in Sweden, 23 managers were excluded due to incomplete contact information. The response rate after two reminders was 23% (n = 295; 104 web-responders and 191 postal responders). The response rate varied across the 21 regions and was generally higher among PCP managers in larger regions and among private PCPs (20% for public PCPs; 25% for private PCPs). The proportion of responses from private PCP-managers (47%) is somewhat higher than available estimates for the target population (42% private PCPs).

20% of the managers who answered the survey had a GP background; 55% had a background as a registered nurse and 25% had another background (physiotherapist, occupational therapist, psychologist, sociologist). As can be noticed in Table 1, GP and non-GP managers had different characteristics and tended to work in different types of PCPs. A majority of GP-managers were males and almost all of them shared time between clinical and administrative duties. A majority of non-GP managers were females and it was less common for non-GP managers to share time between clinical and administrative duties. Comparable estimates regarding managers background for the target population do not exist. According to a survey in 2008 limited to public PCPs, 51% of managers were nurses, 36% of managers were GPs and 13% had another professional background [14]. According to a more recent study, limited to observations from one large Swedish region, but covering both public and private PCPs, 22% of all PCP managers were GPs [12]. A notable difference in regards to differences between GP and non-GP managers in our sample was that GP-managers tended to work in the private sector and for comparatively larger PCPs. GP-managers also perceived the staffing situation of their PCPs as better compared to the average in the region for GPs but not for other staff categories.

**Table 1:** Characteristics of responding managers and their PCPs

	All managers	GP background	Non-GP background
Number of PCPs	295	60	235
Manager characteristics			
Female manager	229 (78%)	21 (35%)	208 (89%)
Manager share time between clinical and administrative duties	135 (46%)	56 (93%)	79 (34%)
Manager with > 5 years at current position	103 (35%)	26 (43%)	77 (33%)
PCP characteristics			
Private PCPs	140 (47%)	40 (67%)	100 (43%)
Size, > 10 000 patients	107 (36%)	27 (45%)	80 (34%)
Staffing situation better than average, doctors	157 (53%)	43 (72%)	114 (49%)
Staffing situation better than average, other staff	192 (67%)	40 (67%)	152 (65%)

### Results from bivariate analysis

Table 2 summarize descriptive results from our bivariate analysis. A statistically significant difference between GP-managers and non-GP managers exists for self-determined goal clarity (higher level for non-GP managers) but not for assigned goal clarity. The lower mean for GP-managers’ self-determined goal clarity is also associated with a higher standard deviation.

**Table 2:** Perceptions of goal clarity, feedback and performance across managers with GP and non-GP background

“Indicate the degree to which the following statements agree with your own opinion”. Likert scale, 1 = Fully disagree, 5 = Fully agree				
	All managers Mean (SD)	GP background Mean (SD)	Non-GP background Mean (SD)	Pearson Correlation^1^
**Goal clarity**				
“The assigned mission from the region is clear, including that the goals to be achieved are well defined”	4,08 (0.861), N = 287	4,07 (0.814), N = 58	4,09 (0.874), N = 227	− 0.009
“The goals that are self-determined at the PCP level are well defined”	3,77 (1.011), N = 288	3,34 (1.132), N = 58	3,87 (0.952), N = 230	− 0.210**
**Feedback messages from region as payer**				
*Information about deviations*				
“Focuses on support and feedback to the PCP to achieve goals such as good medical quality, patient satisfaction, continuity and access”	3,51 (1.052), N = 279	3,02 (1.114), N = 55	3,63 (1.002), N = 224	− 0.233**
“Focuses on support and feedback that helps the PCP to achieve financial results/maintain budget”	2,97 (1.182), N = 278	2,38 (1.225), N = 55	3,11 (1.127), N = 223	− 0.247**
“Provides information on deviations from set guidelines and requirements and shows how we are doing compared to other PCPs”	3,46 (1.066), N = 278	3,06 (1.188), N = 54	3,56 (1.014), N = 224	− 0.187**
*Facilitation of improvement work*				
“Facilitates improvement work through increased knowledge of the PCPs deviations compared to evidence-based knowledge”	2,88 (1.095), N = 263	2,58 (1.184), N = 53	2,95 (1.062), N = 210	− 0.135*
“Facilitates improvement work through increased knowledge based on comparisons and good examples from other PCPs”	3,00 (1.089), N = 275	2,50 (1.194), N = 54	3,12 (1.029), N = 221	− 0.226**
*Quality of data*				
“The data used by the region in its follow-up and feedback is up-to-date and reliable”	3,45 (1.051), N = 267	3,22 (1.254), N = 51	3,50 (0.993), N = 216	− 0.107
“If we implement changes that lead to improvements at the PCP, this is reflected in follow-up and feedback from the region”	2,88 (1.147), N = 267	2,57 (1.233), N = 53	2,96 (1.115), N = 214	− 0.137*
*Social interactions*				
“Stimulates conversations about the PCPs mission and contributes to increased understanding and trust between payers and providers of care”	3,05 (1.086), N = 277	2,67 (1.233), N = 55	3,14 (1.028), N = 222	− 0.174**
**Feedback messages from STRAMA**				
*Information about deviations*				
“Focuses on support and feedback to the PCP to achieve goals such as good medical quality and patient safety”	4,14 (0.843), N = 264	4,06 (0.929), N = 53	4,16 (0.822), N = 211	− 0.047
“Focuses on support and feedback that helps the PCP to achieve appropriate prescribing of drugs”	4,16 (0.809), N = 264	4,17 (0.955), N = 53	4,16 (0.770), N = 211	0.004
“Provides information on deviations from guidelines and shows how we are doing in relation to other PCPs”	4,07 (0.930), N261	4,09 (1.061), N = 53	4,06 (0.896), N = 208	0.014
*Facilitation of improvement work*				
“Facilitates improvement work through increased knowledge of the PCPs deviations compared to evidence-based knowledge”	3,61 (1.032), N = 260	3,62 (1.197), N = 53	3,61 (0.989), N = 207	0.005
“Facilitates improvement work through increased knowledge based on comparisons and good examples from other PCPs”	3,56 (1.023), N = 261	3,34 (1.176), N = 53	3,62 (0.976), N = 208	− 0.109
*Quality of data*				
“The data used in the follow-up and feedback of STRAMA is up-to-date and reliable”	4,33 (0.797), N = 257	4,28 (0.794), N = 53	4,34 (0.799), N = 204	− 0.028
“If we implement changes that lead to improvements at the PCP, this is reflected in follow-up and feedback from STRAMA”	3,96 (0.938), N = 247	3,96 (0.969), N = 52	3,96 (0.933), N = 495	− 0.001
*Social interactions*				
“Stimulates conversations about the work at the PCP and contributes to increased understanding and trust between STRAMA and providers of care”	4,06 (0.872), N = 260	3,94 (0.968), N = 51	4,09 (0.847), N = 209	− 0.068
**Self-rated performance of PCP**				
”Provides care that responds to the preferences of those listed at the PCP”	3,76 (0.764), N = 280	4,04 (0.785), N = 56	3,69 (0.745), N = 224	0.180**
“Provides care responsive to the medical needs of those listed at the PCP”	4,08 (0.674), N = 281	4,27 (0.700), N = 56	4,04 (0.660), N = 225	0.138*
“Provides care in accordance with requirements in the agreement with the region”	4,03 (0.669), N = 283	4,02 (0.757), N = 55	4,03 (0.686), N = 228	− 0.007
**Patient-rated performance (matched data from NPS survey)**				
Overall impression	80,74 (6,99), N = 278	81,94 (5,60), N = 56	80,18 (7,21), N = 222	0.158**

^1^Correlations of perceptions with regard to managers’ professional background. Significance levels: *<0.05, **<0.01; ***<0.001 (2-tailed).

Perceptions of feedback messages varies both depending on type of feedback messages and managers’ background. Feedback messages from STRAMA was more highly rated in all dimensions, irrespective of managers’ background, although a lower mean can be noted for statements focusing on if the feedback facilitated improvement work. The high score for feedback from STRAMA is in contrast to perceptions about feedback messages from the regions as payer. For this feedback, a significant difference also exists depending on managers background in 7 out of 8 statements. Although non-GP managers rated this feedback higher than GP-managers, responses are still substantially lower compared to non-GPs perceptions about feedback from STRAMA. Similar to perceptions about feedback from STRAMA, statements focusing on if the feedback facilitated improvement work received lower means, in particular among GP-managers.

Self-rated responsiveness towards preferences of those listed at the PCP is reported as better among GP-managers. This perception is also in line with scores from patient-rated performance according to the NPS. GP-managers also report higher scores in terms of responsiveness to the medical needs of those listed at the PCP whereas no statistical difference exist when it comes to providing care in accordance with requirements from the region as payer.

### Results from multivariate analysis

Results from OLS regression analysis with patient-reported performance as the dependent variable is presented in Table 3. In the regression analysis, we used two new variables representing overall perceptions of feedback messages based on factor analysis (details from this analysis is reported in Supplementary tables A.2.1-A.2.3 in Appendix 2).

**Table 3:** OLS regression models: variation in patient-assessed performance

	Model 1		Model 2		Model 3	
	Stand. Beta	Sig.	Stand. Beta	Sig.	Stand. Beta	Sig.
Managers’ background (1 = GP)	0.158**	0.008	0.181**	0.002	0.186*	0.037
Goal clarity - self-determined					− 0.078	0.278
Goal clarity – assigned					0.045	0.535
Managers’ background (1 = female)					0.209**	0.009
Managers’ current position (1 = clincal work also)					0.100	0.212
Managers experience (1 = > 5years at current position)					0.092	0.166
Ownership of PCP (1 = private)					0.156*	0.040
Staffing situation, GPs (1 = better than average)					0.162*	0.030
Staffing situation, other staff (1 = better than average)					− 0.051	0.473
Size of PCP (1 = > 10,000 on patient list)					− 0.150*	0.029
A&F payer					− 0.068	0.377
A&F STRAMA					− 0.046	0.538
Self-assessed responsiveness					0.277***	0.000
(Constant)	80.184	0.000	78.081	0.000	71.493	0.000
Control for region	No		Yes		Yes	
Adjusted R2	0.022		0.135		0.353	
N (number of observations)	277		277		183	

Control for region were dummy variables for each of the 21 regions to control for socio-demographic characteristics. No value of tolerance below 0.49 and no value of variance inflation factor (VIF) above 2,18 were observed in the final models. Significance levels: *<0.05, **<0.01; ***<0.001

A significant statistical difference in patient-reported performance depending on managers background existed in all models analysed. Patient-reported performance was significantly better for PCPs managed by GPs. Several other variables contributed with additional explanations behind variation in patient-reported PCP performance. In our final model (3), the adjusted R2 was a reasonable 35%. Having a female background was associated with better PCP performance, as was a private ownership of PCPs, a good staffing situation of GPs and a smaller size of the PCP (negative sign). Patient-reported performance was also significantly associated with managers self-reported performance in terms of responsiveness towards preferences of those listed at the PCP. In contrast to the staffing situation of GPs, there was no statistically significant variation in patient-reported performance with regard to the staffing situation of other staff categories. Similarly, managers’ perceptions of audit and feedback messages from the region as a payer or from STRAMA was not significantly associated with higher or lower patient-reported performance.

## Discussion

Results from the survey support previous findings related to perceptions of feedback messages by managers and health professionals in the context of Swedish primary care. According to a qualitative study with data from focus groups with PCP managers, physicians and other health professionals at seven PCPs, participants did not perceive that feedback messages from regions as payers contributed to improved quality in general [3]. In contrast, feedback from the regional STRAMA group, which was based on clinical data, was often described as meaningful and motivational by professionals. A similar difference in perceptions between feedback messages from the region and STRAMA is also notable in our study. Both GP and non-GP managers had more favourable perceptions of feedback from STRAMA. Feedback from the regions as payer consistently received lower ratings, especially among GP-managers and for statements whether feedback facilitated improvement work and regarding the quality of data in terms of being up-to-date and reliable. Findings related to different perceptions of feedback messages are in line with previous studies in other health care settings. Audit and feedback is more likely to be accepted if professionals trust data, agree with benchmarks and/or consider the clinical topics being audited important [25–27]. Although feedback from STRAMA is received well by both GP and non-GP managers it should be noted that responses to whether it facilitates improvement work is less favourable. As have been noted in other studies, improvement work requires additional capabilities and motivation as well as opportunities to change [3].

The observed differences in perceptions of feedback messages between GP and non-GP managers may have several explanations. A previous qualitative study based on 27 semi-structured interviews with Swedish PCP managers in five regions noted a difference in leadership style between GP and non-GP managers [9]. GP-managers who shared their time between administrative and clinical work seemed to rely more on measures related to clinical quality and patients’ experiences. The argument was that good clinical experience led to satisfied patients, in turn leading to a better working environment, satisfied employees and good financial result. Non-GP-managers, who did not share time between administrative and clinical work to the same extent, seemed to start with volume measures and targets that had to be produced given the resources available, to meet the demands of both payers and patients. The hypothesized differences in leadership style in the previous qualitative study is to some extent supported by results from our survey, and the more favourable view among non-GP managers when it comes to feedback from the region as payer. Results are also in line with previous Swedish studies in both primary and hospital care, that nurse managers are more committed to objectives assigned by regions in comparison with physician managers [10, 14].

In our study we find that GP-managers seem to be more self-confident when rating if care is provided in line with preferences and medical needs of those listed at the PCP, although non-GP managers report a higher score in terms of self-determined goal clarity. A higher self-rated performance is indeed associated with a higher patient-rated performance among PCPs led by GP-managers according to our bivariate analysis. However, the multivariate analysis reveals a more complex relationship. Although higher patient-reported performance correlates with PCP managers being a GP, a positive and significant relationship also exist between patient-reported performance and female managers. We can only speculate about explanations behind this difference at the group level. According to a previous survey of managers in Swedish PC [10] female managers were significantly more loyal to budgetary control and objectives set by the region compared to male managers, also when analysing a sub-sample of GP-managers only.

Similar to findings from several other studies, a larger size of the PCP is associated with lower patient-rated performance [24, 28–30]. Private PCPs correlates with higher patient-reported overall performance, which also confirms results from previous Swedish studies [31]. Not surprisingly, a higher patient-reported performance was strongly linked to PCP characteristics in terms of a better GP staffing situation. However, a similar significant association could not be found for the staffing situation of other staff categories. This difference is potentially important from a policy perspective. At the margin, attempts to improve the staffing situation of non-GPs in Swedish primary care may not increase patient-reported performance if the staffing situation of GPs remain poor.

Interpretation of results from our study need to consider limitations in cross-sectional studies based on survey data. Survey response rate varied across regions and was somewhat lower than expected. For this reason, we are not able to make representative conclusions regarding the total target population of PCP managers. The total number of observations, and similar response rates in different sub-groups, implies that the analysis of differences depending on PCPs and managers characteristics is less sensitive. We also conclude that findings from our survey in several respects echoes findings in previous studies, which support the overall validity. Nevertheless, findings related to differences between GP and non-GPs, and between male and females, may not be valid in other institutional and cultural contexts.

It is important to note that we only analyse correlations and not causality. Both our sample and previous studies of Swedish primary care indicate that the market for PCP managers is somewhat divided between public and private PCPs. Small private PCPs are more likely to be led by GPs, whereas non-GP managers are more common in public PCPs. Although our multivariate analysis should handle these differences, results for sub-groups with fewer observations becomes more sensitive. More importantly, the supply of GP and non-GP managers is influenced by differences in incentives and motivation, creating sorting effects that should be considered when interpreting results. For non-GPs, a transition to a manager position is usually associated with a significant salary increase and an important career step. For GPs, the financial incentive is less clear and being a manager is not necessarily an important career step. These differences, and difficulties of combining clinical and managerial roles, are also referred to when explaining why becoming a manager have been less interesting for Swedish GPs, which is also seen as a problem by the physician union [13]. To sum up, our findings may to some extent reflect that GPs are (more often) less interested in becoming a manager, and therefore more likely to accept being a manager of PCPs with favourable characteristics. In contrast, non-GP managers that have chosen a managerial career do not necessarily have the same option to pick and choose.

Additional limitations of our study are related to the data collected. Inclusion of additional control variables, not least reflecting details of different medical needs and the socioeconomic context at the PCP level, could have contributed to the analysis. The use of registered data on patient-reported performance is a clear strength as results from the OLS regression can be seen as more robust compared to if binary logistic regression using data on self-rated performance had been used. Moreover, systematic differences in self-confidence may exist between both men and females and between GP and non-GP managers that influence self-rated performance. It is also possible that self-rated performance in our study was influenced by the release of results for patient-reported performance ahead of the survey invitation. However, an important limitation when focusing patient-reported performance is that variation in other dimensions of performance, potentially more relevant for feedback messages from the region and STRAMA, is not accounted for. Future studies could incorporate quality indicators that represents additional performance domains as dependent variables, e.g., compliance to existing guidelines for chronic care conditions.

Finally, we only observe managers perception and patient-reported performance at a certain point in time. The survey was also ignorant of managers capabilities in terms of initiating and implementing change. It is possible that important differences in this respect exists between GP and non-GP managers, and to identify these would require a more longitudinal study design. As suggested by a previous study [10], non-GP managers may have less autonomy from owners of PCPs and experience more problems when attempting to implement changes. A related perspective, and opportunity for additional research, is possible differences between managers with different professional background´s as perceived by PCP staff.

## Conclusions

Both GP and non-GP managers rated the quality and support of feedback messages from professional committees higher than feedback from regions as payers. Differences in perceptions were especially striking among GP-managers. Results from OLS regression suggest that GP-managers correlate with better patient-reported PCP performance when controlling for PCP and other managerial characteristics. A significant positive relationship with patient-reported performance was also found for female managers, a smaller size of the PCP and a good staffing situation of GPs. As reversed causality cannot be excluded, the findings may to some extent reflect that GPs are more likely to accept being a manager of a PCP with favourable characteristics.

## Electronic supplementary material

Below is the link to the electronic supplementary material.

Supplementary Material 1

Supplementary Material 2

## Data Availability

The datasets analysed during the current study are available from the corresponding author on reasonable request.
