# Supplementary material for: Better with GPs as managers? – Variation in perceptions of feedback messages, goal-clarity and performance across manager´s in Swedish primary care
Source: BMC Health Serv Res. 2023 Jun 14;23:639. doi: 10.1186/s12913-023-09586-2 (PMC10268428; doi:10.1186/s12913-023-09586-2)
Supplement: Supplementary file 1 — Supplementary Material 1 [file 12913_2023_9586_MOESM1_ESM.docx]

**Appendix 1** – Survey questions and response alternatives (translation from original Swedish language)

| **Survey to managers** |  |
| --- | --- |
| **Characteristics of manager and PCP** | Anchors |
| What is your professional background?)  If “other”, please specify  Gender  In your current position, do you share your time between administrative and clinical work  How many years of experience do you have at your current position?  Ownership  Size  In relation to other PCPs in the region, how is your staffing situation regarding GPs  In relation to other PCPs in the region, how is your staffing situation regarding other staff | GP/RN/Other  Free text  Male/female/other  Yes/No  No of years  Private/public  (<5k, 5-10k,10-15k, >15k)  Likert scale, 1=much worse than average, 5=much better than average) |
| **Audit & feedback from the region (payer of services)** |  |
| “Indicate the degree to which the following statements agree with your own opinion”:  1. Focuses on support and feedback to the PCP to achieve goals such as good medical quality, patient satisfaction, continuity and access  2. Focuses on support and feedback that helps the PCP achieve financial results/maintain budget  3. Provides information on deviations from set guidelines and requirements and shows how we are doing compared to other PCPs  4. Stimulates conversations about the PCPs mission and contributes to increased understanding and trust between payers and providers of care  5. Facilitates improvement work through increased knowledge of the PCPs deviations compared to evidence-based knowledge  6. Facilitates improvement work through increased knowledge based on comparisons and good examples from other PCPs  7. The data used by the region in its follow-up and feedback is up-to-date and reliable  8. If we implement changes that lead to improvements at the PCP, this is reflected in follow-up and feedback from the region | 1 = Fully disagree  5 = Fully agree |
| **A&F from STRAMA (professional committees)** |  |
| “Indicate the degree to which the following statements agree with your own opinion”:  1. Focuses on support and feedback to the PCP to achieve goals such as good medical quality and patient safety  2. Focuses on support and feedback that helps the health center achieve appropriate prescribing of drugs  3. Provides information on deviations from guidelines and shows how we are doing in relation to other PCPs  4. Stimulates conversations about the work at the PCP and contributes to 5. increased understanding and trust between STRAMA and providers of care  5. Facilitates improvement work through increased knowledge of the PCPs deviations compared to evidence-based knowledge  6. Facilitates improvement work through increased knowledge based on comparisons and good examples from other PCPs  7. The data used in the follow-up and feedback of STRAMA is up-to-date and reliable  8. If we implement changes that lead to improvements at the PCP, this is reflected in follow-up and feedback from STRAMA | 1 = Fully disagree  5 = Fully agree |
| **Goal clarity** |  |
| “Indicate the degree to which the following statements agree with your own opinion”:  The assigned mission from the region is clear, including that the goals to be achieved are well defined  The goals that are self-determined at the PCP level are well defined | 1 = Fully disagree  5 = Fully agree |
| **Self-rated performance** |  |
| “Rate the following aspects of your PCP in comparison with other PCPs in your region”:  Provides care that responds to the preferences of those listed at the PCP  Provides care responsive to the medical needs of those listed at the PCP  Provides care in accordance with requirements in the agreement with the region | 1=Significantly below average  5= Significantly above average |
| **Survey to patients** |  |
| **Overall impression** |  |
| Did you find that the atmosphere at the PCP was good?  Do you feel that your needs for healthcare/treatment have been met satisfactorily?  Would you recommend the PCP to anyone else in your situation? | 1 = No, not at all  5 = Yes, completely |
