# Supplementary material for: Better with GPs as managers? – Variation in perceptions of feedback messages, goal-clarity and performance across manager´s in Swedish primary care
Source: BMC Health Serv Res. 2023 Jun 14;23:639. doi: 10.1186/s12913-023-09586-2 (PMC10268428; doi:10.1186/s12913-023-09586-2)
Supplement: Supplementary file 2 — Supplementary Material 2 [file 12913_2023_9586_MOESM2_ESM.docx]

**Appendix 2. Results from factor analysis.**

**Supplementary table A.2.1.** Correlation matrix: A&F from the region (payer of services).

|  | Statement 1 | Statement 2 | Statement 3 | Statement 4 | Statement 5 | Statement 6 | Statement 7 |
| --- | --- | --- | --- | --- | --- | --- | --- |
| Statement 2 | .643** |  |  |  |  |  |  |
| Statement 3 | .496** | .451** |  |  |  |  |  |
| Statement 4 | .564** | .590** | .503** |  |  |  |  |
| Statement 5 | .537** | .589** | .555** | .643** |  |  |  |
| Statement 6 | .555** | .630** | .534** | .627** | .796** |  |  |
| Statement 7 | .377** | .440** | .362** | .432** | .479** | .476** |  |
| Statement 8 | .533** | .525** | .464** | .610** | .659** | .623** | .567** |

** Correlation (Pearson) is significant at the 0.01 level (2-tailed). See appendix 1 for formulation of statements about A&F from the region.

**Supplementary table A.2.2.** A&F from STRAMA (professional committees).

|  | Statement 1 | Statement 2 | Statement 3 | Statement 4 | Statement 5 | Statement 6 | Statement 7 |
| --- | --- | --- | --- | --- | --- | --- | --- |
| Statement 2 | .744** |  |  |  |  |  |  |
| Statement 3 | .591** | .636** |  |  |  |  |  |
| Statement 4 | .649** | .629** | .655** |  |  |  |  |
| Statement 5 | .579** | .614** | .616** | .622** |  |  |  |
| Statement 6 | .557** | .593** | .593** | .670** | .800** |  |  |
| Statement 7 | .565** | .527** | .585** | .626** | .503** | .476** |  |
| Statement 8 | .601** | .610** | .513** | .630** | .621** | .600** | .667** |

** Correlation (Pearson) is significant at the 0.01 level (2-tailed). See appendix 1 for formulation of statements about A&F from STRAMA.

**Supplementary table A.2.3.** Factor analysis of constructs – A&F from the region and A&F from STRAMA.

| **A&F from the region** |  |
| --- | --- |
| Statement 1 | .700 |
| Statement 2 | .748 |
| Statement 3 | .660 |
| Statement 4 | .793 |
| Statement 5 | .873 |
| Statement 6 | .856 |
| Statement 7 | .571 |
| Statement 8 | .758 |
| Eigenvalue | 4.932 |
| Variance explained | 61.650 |
| Cronbach's alpha (standardized) | .909 |
| KMO sampling adequacy | .908 |
| Bartlett's test of sphericity | .000 |
| **A&F from STRAMA** |  |
| Statement 1 | .809 |
| Statement 2 | .813 |
| Statement 3 | .757 |
| Statement 4 | .837 |
| Statement 5 | .800 |
| Statement 6 | .792 |
| Statement 7 | .707 |
| Statement 8 | .767 |
| Eigenvalue | 5.321 |
| Variance explained | 66.516 |
| Cronbach's alpha (standardized) | .928 |
| KMO sampling adequacy | .899 |
| Bartlett's test of sphericity | .000 |
| KMO sampling adequacy | .899 |
| Bartlett's test of sphericity | .000 |
